# Is a non-synonymous SNP in the *HvAACT1* coding region associated with acidic soil tolerance in barley?

**DOI:** 10.1590/1678-4685-GMB-2016-0225

**Published:** 2017-05-08

**Authors:** Jéssica Rosset Ferreira, Bruna Franciele Faria, Moacyr Comar, Carla Andréa Delatorre, Euclydes Minella, Jorge Fernando Pereira

**Affiliations:** 1 Departamento de Plantas de Lavoura, Faculdade de Agronomia, Universidade Federal do Rio Grande do Sul, 91501-970, Porto Alegre, RS, Brazil; 2 Programa Multicêntrico de Pós-Graduação em Bioquímica e Biologia Molecular, Universidade Federal de São João del-Rey, 35501-296, Divinópolis, MG, Brazil; 3 Embrapa Trigo, 99001-970, Passo Fundo, RS, Brazil; 4 Embrapa Gado de Leite, 36038-330, Juiz de Fora, MG, Brazil

**Keywords:** Aluminium tolerance, citrate transporter, haplotype, Hordeum vulgare, single nucleotide polymorphism

## Abstract

The barley *HvAACT1* gene codes for a citrate transporter associated with tolerance to acidic soil. In this report, we describe a single nucleotide polymorphism (SNP) in the *HvAACT1* coding region that was detected as T-1,198 (in genotypes with lower root growth on acidic soil) or G-1,198 (greater root growth) and resulted in a single amino acid change (L/V-172). Molecular dynamic analysis predicted that HvAACT1 proteins with L or V-172 were stable, although the substitution led to structural changes within the protein. To evaluate the effect of the SNP on tolerance to acidic soil, barley accessions were separated into haplotypes based on the presence of a 1 kb insertion in the *HvAACT1* promoter and a 21 bp insertion/deletion. These markers and the SNP-1,198 allowed the identification of five haplotypes. Short-term soil experiments showed no difference in root growth for most of the accessions containing the 21 bp insertion and T or G-1,198. In contrast, genotypes showing both the 21 bp deletion and G-1,198, with one of them having the 1 kb insertion, showed greater root growth. These results indicate that the SNP was not advantageous or deleterious when genotypes from the same haplotype were compared. The occurrence of the SNP was highly correlated with the 21 bp insertion/deletion that, together with the 1 kb insertion, explained most of the barley tolerance to acidic soil.

## Introduction

The toxic trivalent aluminium cation (Al^3+^) occurs at higher concentration in acidic soils (pH < 5.0) (Kinraide, 1991). When present in soil, Al^3+^ affects a number of plant cellular functions through various intra- and extracellular interactions that result in lower root growth (Ryan and Delhaize, 2010). Plants with shorter roots have a lower uptake of water and nutrients and are more easily affected by stressful conditions such as diseases, drought and heat, all of which can potentially reduce plant development and yield. Different mechanisms have evolved to allow plants to deal with harmful Al^3+^ concentrations, the most studied of these being the release of organic anions by the root apex. Various plant genes related to organic acid transporters have been isolated (Ryan *et al.*, 2011), thereby allowing a more detailed study of their function.

The *HvAACT1* gene (Furukawa *et al.*, 2007), also known as *HvMATE* (Wang *et al.*, 2007), codes for a membrane transporter that is a member of the multidrug and toxic compound family responsible for citrate efflux in barley. When released by the root apex into soil, citrate forms a complex with Al^3+^, thereby reducing the toxicity of this metal and allowing greater root growth (Ryan and Delhaize, 2010). The release of citrate by root apices is therefore one mechanism of Al^3+^ tolerance in barley. The level and location of *HvAACT1* expression is highly correlated with Al^3+^ tolerance. For instance, the transgenic expression of *HvAACT1* using a strong and constitutive promoter increases barley Al^3+^ tolerance (Zhou *et al.*, 2013). In addition, when a 1 kb insertion is detected upstream of the *HvAACT1* coding region, not only is the expression enhanced but it also switches to the root apex, thereby altering the primary function of this gene, which is the release of citrate to the xylem to facilitate iron translocation (Fujii *et al.*, 2012). This 1 kb insertion occurs only in Al^3+^-tolerant genotypes of cultivated barley (*Hordeum vulgare* spp. *vulgare*) from East Asia where acidic soils are prevalent (Fujii *et al.*, 2012). The higher expression of the *HvAACT1* gene in the root apex is correlated with the amount of citrate released by the barley root tip which is, ultimately, correlated with the Al^3+^ tolerance (Zhao *et al.*, 2003; Wang *et al.*, 2007; Fujii *et al.*, 2012). Another important variation, not found in the *HvAACT1* coding region, is a 21 bp insertion/deletion detected by the marker HvMATE-21indel (Bian *et al.*, 2013). This marker is located in the 3’ non-translated region of the *HvAACT1* gene and the 21 bp deletion is associated with Al^3+^ tolerance. The HvMATE-21indel marker reportedly explains from 66.9% to 71% of the variation for the acidic soil tolerance in barley (Bian *et al.*, 2013; Ma *et al.*, 2016). Both the 1 kb insertion at the *HvAACT1* promoter and the HvMATE-21indel marker can be easily detected by PCR, thus allowing the characterization of barley accessions.

There is variation in the *HvAACT1* gene sequence among barley genotypes (Furukawa *et al.*, 2007; Bian *et al.*, 2015), with one single nucleotide polymorphism (SNP) occurring per 29 bp of this gene (Bian *et al.*, 2015). This frequency is higher than that of one SNP per 240 bp reported for barley in general (Duran *et al.*, 2009). SNPs also occur in organic acid transporter genes from wheat (Sasaki *et al.*, 2004; Tovkach *et al.*, 2013), with one of them that is present in the *TaMATE1B* promoter being responsible for a two-fold increase in gene expression (Tovkach *et al.*, 2013). SNPs can result in important phenotypic alterations, especially when they occur in regulatory and coding regions, where the SNP can lead to an amino acid change (non-synonymous SNP). In barley, non-synonymous SNPs have been correlated with the uzu phenotype (Chono *et al.*, 2003) and have also been identified in the *Isa* gene (Bundock and Henry, 2004). In addition, SNPs have been detected in the *Bmy1* gene coding for β-amylase (Zhang *et al.*, 2007), in the *Rrs2* gene that confers resistance to leaf scald (Hanemann *et al.*, 2009) and in the *sdw1/denso* gene that controls plant height, yield and quality (Jia *et al.*, 2009). Indeed, SNPs within individual barley genes appear to be more frequent than previously reported and the level of useful SNP-derived markers in barley is estimated to be greater than in wheat (Shavrukov, 2016).

The search for variations of the *HvAACT1* gene is particularly important because potentially new markers, mechanisms and/or ways of regulation can be found. For instance, two new *HvAACT1* alleles were recently reported, although the mechanism of Al^3+^ tolerance was similar to that previously described (Bian *et al.*, 2015; Ma *et al.*, 2016). The importance of analyzing this gene is related to the fact that barley is one of the most Al^3+^-sensitive plant species among the common cereals. In addition, the domestication of cultivated barley led to a genetic bottleneck with a less polymorphic genetic pool when compared to wild barley (*Hordeum vulgare* ssp. *spontaneum*) (Kilian *et al.*, 2006) and barley breeding programs have experienced loss of genetic diversity in modern cultivars (Ferreira *et al.*, 2016). This can contribute to lower polymorphism for an important trait such as Al^3+^ tolerance. The potential for improving barley Al^3+^ tolerance through recombination of genotypes, the tolerance of which is controlled by the same gene, is very low and different gene sources should be evaluated (Minella and Sorrells, 2002). These limitations mean that there is a need to search for new gene variations to overcome the low variability for some traits in barley and enhance the tolerance to stress.

In this context, the aim of this study was to examine the relationship between genetic variation in the *HvAACT1* gene and barley root growth on acidic soil. Initially, we identified a non-synonymous SNP that resulted in one amino acid change and was correlated with larger root growth on acidic soil. We then used molecular dynamics to investigate the effect of this change on the HvAACT1 protein. Based on two markers linked to the *HvAACT1* gene, we separated different genotypes into haplotypes and representatives of the haplotypes were investigated in a short-term soil experiment. This haplotype-based phenotyping excluded the influence of other regions, thereby allowing us to evaluate only the impact of the SNP on barley root growth on acidic soil.

## Material and Methods

### Genotypes

In this study, we evaluated the 76 barley (*Hordeum vulgare* ssp. *vulgare*) genotypes listed in Table 1. All seeds were provided by the Embrapa Trigo active germplasm bank.

**Table 1:** *HvAACT1* haplotypes identified in 76 cultivated barley genotypes based on two markers linked in the *HvAACT1* gene and SNP-1,198.

Genotypes	1 kb insertion*	HvMATE-21indel**	SNP-1 198#	Residue 172
Dayton, Murasakimochi, Nakano Wase, and Sunrise.	Present	Deletion	G	Valine
Antarctica 01, Antarctica 6, Barlena, Botnia, BRS Borema, Cheri, Chevron, Contesse, Corniche, Dorett, FM 404, FM 420, FM 438, Gold, IPB 1219, Ismene, Krystal, Lenka, Meltan, MN 577, MN 607, MN 642, MN 643, MN 668, MN 682, Natasha, New Golden, Novosadski 163, Novosadski 301, Omugi, Pacific, Pernilla, Princesse, Prisma, Recla 1, Recla 44, Tallon, Triumph, Ulandra, Villa and WIR 24724.	Absent	Deletion	G	Valine
Onslow, Strada, Windich and Yagan.	Absent	Insertion	G	Valine
Camelot, Clark, Egypt, Gainesville 1, Glenn, Harrington, Jarek, Kasota, Kawa Mizuki, Kawa Saigoku, Klaxon, Lion, Madonna, MN 6021, Orbit, Paraí-I, Pyramid, Recla 122, Recla 92, Ricci, Target, Toga, Valentine, Vaughn, Waranga and Zapata.	Absent	Insertion	T	Leucine
Atlas 57.	Absent	Insertion	T/G	-

* 1 kb insertion in the *HvAACT1* promoter as described by Fujii *et al.* (2012);

** 21 bp insertion/deletion in the 3’ non-translated region of the *HvAACT1* gene as described by Bian *et al.* (2013);

# SNP at position 1,198 identified here by sequencing and the residue 172 identified by translation of the sequence.

### Evaluation of root growth in a short-term soil experiment

The root length after seven days of growth on acidic and limed soil was used to evaluate the Al^3+^ tolerance of barley. Acidic soil (pH 4.2 measured in water) in which the exchangeable aluminium represented 78.9% of the effective cation exchange capacity, was collected at a depth of 0-20 cm from the Embrapa Trigo experimental area (28,216979S, 52,408428W). Preliminary tests showed that barley genotypes known to differ in Al^3+^ tolerance could not be clearly discriminated after growing in that soil. In this way, two concentrations of CaCO_3_ were used to lime the soil increasing the pH to 4.7 and 5.9; this in turn reduced the soluble Al^3+^ to 48.3% and 1.5%, respectively. These two soils were used as acidic (pH 4.7) and limed (pH 5.9) soil in the short-term experiments. The acidic and limed soil (450 g each) were distributed in pots (5 cm in diameter and 25 cm height) and irrigated to achieve 90% of the field capacity. The experiment was done in duplicate biological samples, each of which consisted of 4-5 germinated seeds with a root length between 0.5 and 1.0 cm for each genotype. The germinated seeds were transferred to pots containing limed soil (control plants) or acidic soil (treated plants) and incubated in a glasshouse at 16°C/22°C (night-day) with natural light. The pots were watered every two days to maintain the same level of field capacity. After seven days, the roots were carefully removed from the soil and the length of the longest root was measured. The relative root length (RRL) was estimated as (root length on acidic soil/root length on limed soil) x 100. Errors associated with deriving the RRL were calculated as SE_RRL_= RRL [(SE_*x*_/*x*)^2^+ (SE_*y*_/*y*)^2^]^1/2^ where *x* represents the mean root length on limed soil and *y* the mean root length on acidic soil.

### Statistical analysis

The data related to the root growth measurements were expressed as mean RRL ± SE_RRL_. The statistical comparisons were based an overlapping confidence limits approach detailed previously (Zhou *et al.*, 2013) with p < 0.05 indicating significance.

### DNA extraction

Five seeds of each genotype were surface sterilized and pre-germinated in the dark at 23°C for up to two days, after which they were transferred to 50 mL plastic cups containing a mixture of soil, vermiculite and sand (1:1:1) and placed on a laboratory bench under ambient light and irrigated every two days. After one week, the leaves were cut, transferred to a 2 mL Eppendorf tube containing three stainless steel beads (2.3 mm in diameter), frozen in liquid nitrogen and triturated in a Mini-Beadbeater^TM^ (Biospec Products) for 1 min. Total DNA was extracted using a CTAB-based protocol (Doyle and Doyle, 1987) and quantified on 0.8% agarose gels.

### *HvAACT1* sequencing and sequence analysis

The cultivars Antarctica 01, FM 404, MN 6021 and Paraí-I were selected for sequence analysis of the *HvAACT1* gene. These genotypes differ in root growth on acidic soil, with Antarctica 01 and FM 404 showing greater root growth compared to MN 6021 and Paraí-I (Ferreira, 2015). DNA from these genotypes was amplified using various primers to obtain three overlapping fragments corresponding to 1,254 bp of the beginning of the gene (first fragment), 1,222 bp of the middle of the gene (second fragment) and 1,242 bp of the end of the gene (third fragment) (Figure S1). The primers used to obtain the three fragments were CTCTCATCCCTCCTCTCACG and TTCTCAAGGTCTTGGCTGCT for the first fragment, TCTGTATCTACCCGCTTGTTAGC and GACGCCAGAGTGACACAGAA for the second fragment and TGGCTCTGAAAATGCTCTGTT and TCACTTCCGGAGGAAAACCCA for the third fragment. The amplification reaction contained 1 x PCR buffer, 0.25 mM of each dNTP, 1 x Q solution, 10 μM of each primer, 1 unit of *Taq* DNA polymerase (Qiagen) and 150 ng of total DNA. The amplification program consisted of 40 cycles at 94 °C for 30 s, 55 °C for 30 s and 72 °C for 1.5 min followed by a final step of 72 °C for 10 min. PCR fragments were cloned in the pGEM-T Easy vector (Promega). After transforming *E. coli*, the plasmids were extracted using the Wizard Plus SV Minipreps DNA purification system (Promega). Plasmid quantification was done using Nanodrop (Thermo Scientific). The plasmids were sequenced using primers T7 promoter (TAATACGACTCACTATAGGG), SP6 (ATTTAGGTGACACTATAG), HvMATE3 (GGTTGGATGGTCGTGAGATT), HvMATE5 (GATCCCCTGGCTTCCTTG), HvMATE6 (TGTCAGCAAAGGTGAAAAATTC) and HvMATE7 (GTATCGGTCGCTTGATTTGG) for the first fragment, T7, SP6, HvMATE10 (ATGTTTCACACCCATGATGC), HvMATE11 (CTAGTTCAGGCCGTGTTCCT), HvMATE12 (CCATGTGGCAGACAAACATC) and HvMATE13 (CCTTACAATTTCTTTTGCAGTGG) for the second fragment, and T7, SP6, HvMATE16 (GCAAAGAGAAAGAGGTCACCA), HvMATE17 (TCTCGTGTTCTGCAGGTTTG), HvMATE18 (CTCGGGACAAGTTTCAGAGC) and HvMATE19 (CATCAACTTCGGAGCACAAG) for the third fragment. Sequencing reactions were done using 200-500 ng of each plasmid, 3.2 μM of primer, sequencing buffer and Big Dye Terminator version 3.1. The thermocycling conditions were 96 °C for 1 min, followed by 35 cycles at 96 °C for 20 s, 56 °C for 20 s and 60 °C for 2 min and 45 s. After amplification, the reactions were precipitated, resupended in Hi-Di formamide, denaturated and run on an ABI 3130xl sequence analyzer. Sequencing Analysis Software version 5.1.1 and the Staden sequencing analysis package (Staden, 1996) were used to analyze the sequences. The four sequences obtained here were deposited in GenBank with the following accession numbers: KX278713 (Antarctica 01), KX278714 (FM404), KX278715 (MN 6021) and KX278716 (Paraí-I). Alignments of the *HvAACT1* gene and protein sequences were done with the ClustalW program (Thompson *et al.*, 1994).

### Construction of models and molecular dynamics of HvAACT1 proteins

Based on the primary sequence of the HvAACT1 protein from the Al^3+^-tolerant genotype Murasakimochi (GenBank BAF75822) and the Al^3+^-sensitive genotype Haruna Nijo (GenBank BAF75823), two transporters were constructed with MODELLER 9.15 software (Eswar *et al.*, 2006) using multiple templates extracted from the Protein Data Bank (PDB). The templates used for BLAST alignment were 4MLB, 4LZ6, 3VVN, 3W4T and 3MKT; all of them are ionic transporters belonging to the MATE family. Five models were generated for each transporter and all models presented structural similarity and DOPE (Discrete Optimized Protein Energy) score values less than -5.7 x 10^-4^ (Shen and Sali, 2006), which is the tolerance value for use of the model. Of the five models, only the one with the lowest DOPE score was used. The molecular dynamic simulation was done with the GROMACS 5.0.5 package (Abraham *et al.*, 2016) using the force field Amber99SB-ILDN (Lindorff-Larsen *et al.*, 2010). The proteins were solvated with TIP3P model for water molecules. To avoid a possible clash of some atoms during construction of the models, the structure was searched for such spurious contacts between atoms that were then eliminated. This process (known as energy minimization) was applied using the method of steeped descent. To equilibrate the minimized system, two restriction dynamics were run: one in the NVT ensemble (where the number of particles, volume and temperature were kept constant) and another in the NPT ensemble (where volume was changed for pressure). The temperature was held at 310 K by a Nosé-Hoover thermostat (Hünenberger, 2005) and the pressure at 1 atm by a Parrinello-Rahman barostat. Trajectories were produced using the NPT ensemble with 20 ns of simulation and a time step of 0.8 fs for each system.

### Amplification of two markers linked to the *HvAACT1* gene

DNA from the 76 barley genotypes was amplified using the 1 kb insertion and HvMATE-21indel as markers. The 1 kb insertion marker (GGTCCAACACTCTACCCT TCCTT and GGTGCGAGTTGCCCCTAGCTATTACA GA) (Fujii *et al.*, 2012) was amplified in reactions with a final volume of 20 μL containing 1 x PCR buffer, 0.25 mM of each dNTP, 1 x Q solution, 10 μM of each primer, 1 unit of *Taq* DNA polymerase (Qiagen) and 150 ng of total DNA. The reactions were incubated in a PTC-100 thermal cycler (MJ Research) programmed as follows: 94 °C for 3 min, then 45 cycles at 94 °C for 30 s, 60 °C for 30 s and 72 °C for 2 min and a final step at 72 °C for 5 min. The amplicons were separated on 1% agarose gels (Figure S2) with a 1,844 bp fragment corresponding to the presence of a 1 kb insertion in the *HvAACT1* promoter and an 821 bp fragment indicating its absence. The HvMATE-21indel marker (GCTAGGGCTTGAAAACTGTTTG and GACGAACTG TACGATGATGATGC) (Bian *et al.*, 2013) was amplified in reactions with a final volume of 20 μL containing 1x PCR master mix (Promega), 10 μM of each primer and 100 ng of total DNA. The reactions were run in a PTC-100 thermal cycler (MJ Research) programmed as follows: 94 °C for 3 min, then 40 cycles at 94 °C for 30 s, 55 °C for 30 s and 72 °C for 40 s and a final step at 72 °C for 5 min. The PCR fragments were separated on 2% agarose gels (Figure S2) with a 497 bp amplicon corresponding to the 21 bp deletion and an 518 bp fragment to the 21 bp insertion.

### SNP detection

Primers Cit7F (GCAGCCAAGACCTTGAGAAAGC) and Cit7R (GCCTGAACTAGCCCGAGAAATG) (Bian *et al.*, 2015) were used to amplify DNA from the barley genotypes. The amplification reaction contained 1 x buffer, 2.5 mM MgCl_2_, 0.35 mM of each dNTP, 0.2 μM of each primer, 0.75 U of *Taq* polymerase (RBC Bioscience) and 150 ng of total DNA. The amplification program consisted of one step at 94 °C for 3 min followed by 40 cycles at 94 °C for 30 s, 55 °C for 30 s and 72 °C for 40 s and a final step at 72 °C for 5 min. After thermocycling, the samples were precipitated and checked on a 0.8% agarose gel. Two microliters of the precipitated PCR fragment were used directly in the sequencing reaction with primer Cit7R (3.2 μM), sequencing buffer and Big Dye Terminator version 3.1. Each sample was sequenced twice in the thermocycling conditions described above. SNP-1,198 was detected by considering the reverse complementary sequence (Figure S2).

## Results

### Detection of an SNP in the *HvAACT1* gene correlated with barley Al^3+^ tolerance

**Figure 1:**
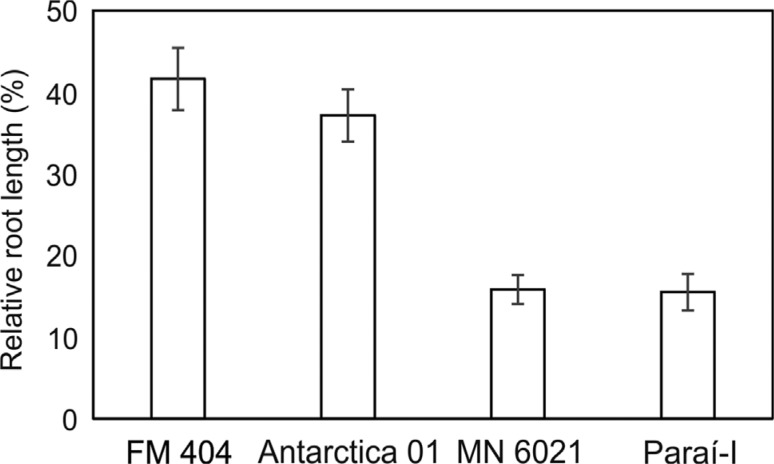
Relative root length (RRL) of four barley genotypes in relation to Al^3+^ tolerance in a short-term soil experiment. The RRL was calculated as the percentage of root length in acidic soil relative to root length in limed soil. The columns represent the mean RRL ± SE_RRL_.

Four barley cultivars (Antarctica 01, FM 404, MN 6021 and Paraí-I) were selected for the analysis of Al^3+^ tolerance in short-term soil experiments. The relative root length (RRL) of these four genotypes was determined after seven days of growth on acidic (pH 4.7) and limed (pH 5.9) soil (Figure 1). Antarctica 01 and FM 404 showed greater RRL than Paraí-I and MN 6021, indicating a clear difference in Al^3+^ tolerance. Sequence alignment of the *HvAACT1* gene in these four cultivars (Figure S3) allowed the identification of seven SNPs spread across intron 10 (two SNPs), exon 8 (two SNPs) and exons 3, 4 and 11 (one SNP each). The most frequent change was between C/T (four SNPs), followed by G/A (two SNPs) and T/G (one SNP). Among the five SNPs detected in exons, only one resulted in an amino acid change in the HvAACT1 protein (Figure S4) and was correlated with Al^3+^ tolerance. This change occurred in exon 3 when a thymidine (T-1,198), present in genotypes MN 6021 and Paraí-I that showed lower root growth on acidic soil, was replaced by a guanine (G-1,198) in cultivars Antarctica 01 and FM 404 that showed greater root growth. This mutation resulted in leucine (L-172) present in the HvAACT1 protein from MN 6021 and Paraí-I being changed to valine (V-172) in Antarctica 01 and FM 404. Comparison of the alignment of the HvAACT1 protein with other genotypes of known Al^3+^ tolerance (Al^3+^-tolerant genotype Murasakimochi and Al^3+^-sensitive genotype Haruna Nijo) showed that the amino acid change (L/V-172) was still associated with Al^3+^ tolerance (Figure S4). This finding indicated that SNP-1,198 was correlated with barley Al^3+^ tolerance.

### Molecular dynamic analysis of HvAACT1 proteins

**Figure 2:**
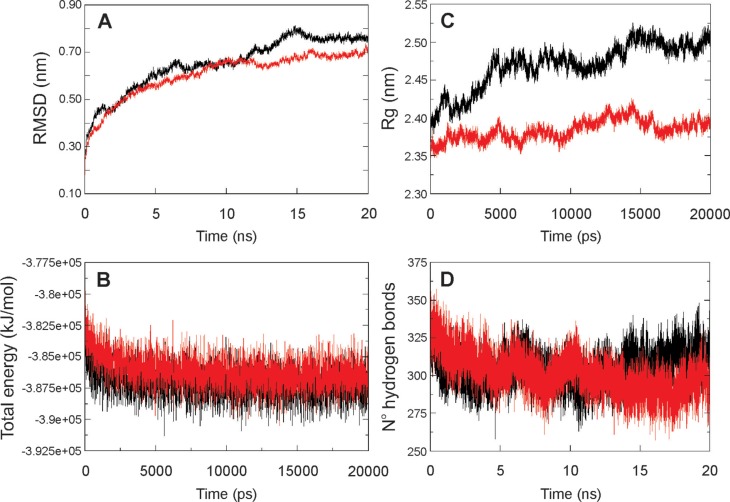
Analysis of two HvAACT1 proteins that differed only at residue 172. Red lines indicate the HvAACT1 protein with L-172 and black lines indicate the protein with V-172. The panels show the results for the root mean square deviation **(A)**, total energy **(B)**, radius of gyration **(C)** and number of hydrogen bonds **(D)**.

**Figure 3:**
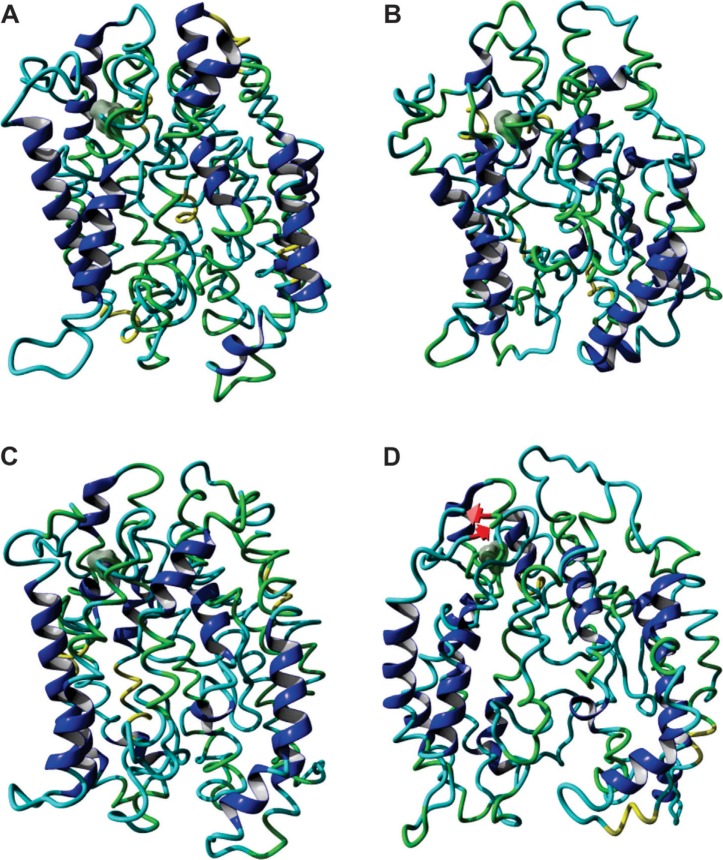
Molecular models of the HvAACT1 proteins that differed only at residue 172, namely, L-172 **(A,** **B)** and V-172 **(C,** **D)**. The left panels refer to the situation at the start of the dynamic analysis and the right panels refer to the average structure obtained during the dynamic analysis (20 ns). Grey cloud represents the position of residue 172.

To evaluate the impact of the L/V-172 mutation, two systems were constructed and simulated by molecular dynamic methods. One system was based on the HvAACT1 protein from an Al^3+^-sensitive genotype (Haruna Nijo) and the other was based on the same protein but from an Al^3+^-tolerant genotype (Murasakimochi). The two proteins differed only in residue 172 (L or V). The RMSD (root mean square deviation) for the two transporters during the 20 ns of dynamic showed that the change in residue 172 did not significantly modify the structural behaviour of the proteins (Figure 2A). This was because the increase in the RMSD was common to both proteins and could be attributed to the lack of a bilayer membrane that restricts movement of the transporters’ structures. The two systems were also energetically stable (Figure 2B). On the other hand, the radius of gyration (Rg), which measures the compactness of a system during the course of molecular dynamic simulation, differed between the two proteins. The protein with L-172 showed only small fluctuation during simulation (variation of ~0.09 nm), indicating that the protein structure remained compact, while the protein with V-172 showed larger fluctuation (variation of 0.19 nm) (Figure 2C). This difference can be explained by modifications at the bottom of the protein that remains outside the membrane (Figure 3 and S5) and could be related to differences in citrate transport. Figure 2D shows a reduction in the number of hydrogen bonds (Hb) in both proteins during the simulation. However, the protein with V-172 showed a tendency to reconnect these bonds despite the similar average number of Hb in the two proteins (304.43 ± 11.02 Hb for V-172 and 301.51 ± 13.15 for L-172). The decrease in Hb shown by the HvAACT1 protein with L-172 was attributable to deformation in the structure (as shown in Figure 2C).

The single change at residue 172 led to important structural changes within the HvAACT1 protein. The transporter with L-172 was more compact when compared to the protein with V-172, which was more open at the lower end (right panels in Figure 3). The protein with L-172 showed little change in its structure during the dynamic when compared to the starting structure (Figures 3A,B and S5). The transporter with V-172 (in genotypes with greater root growth on acidic soil) showed considerable relaxation in the starting structure that could facilitate the passage of citrate across the transporter, leading to faster release thereof (Figures 3C,D and S5).

The analysis in Figure 2 shows that both systems were stable throughout the dynamic analysis and that the duration of this simulation was satisfactory for this work. We conclude that residue 172 is important for maintaining the HvAACT1 transporter structure because this single mutation led to considerable changes during the dynamic analysis of these two proteins (Figure S5).

### Allelic variability in the 1 kb insertion and HvMATE-21indel marker

Since the molecular dynamic analysis revealed that a change in residue 172 was important for transporter structure, we were interested in evaluating the impact of that change on root growth in a larger collection of barley genotypes. To assess the impact of the L/V-172 modification precisely, the barley genotypes were separated into haplotypes based on the combination between the alleles of two markers, the 1 kb insertion and HvMATE-21indel, linked to the *HvAACT1* gene. This strategy was important because greater root growth could be linked to the presence of favourable alleles for one or both markers rather than to the SNP itself. For instance, the four genotypes (Antarctica 01, FM 404, Paraí-I and MN 6021), whose root growth was previously associated with the SNP (Figure 1), also showed polymorphism for the HvMATE-21indel marker (Table 1). In this context, the better root growth shown by Antarctica 01 and FM 404 could be associated with the G-1,198 and/or with the 21 bp deletion (497 bp amplicon) of the HvMATE-21indel marker.

We detected two alleles for both markers. The 1 kb insertion resulted in the amplification of 1,844 bp (presence of the insertion in the *HvAACT1* promoter region) or 821 bp (absence of the insertion) fragments while the HvMATE-21indel marker yielded 518 bp or 497 bp amplicons related to the 21 bp insertion or 21 bp deletion, respectively (Figure S2). Based on the combination of these alleles and the SNP at position 1,198, five haplotypes were identified. In one genotype (Atlas 57), the sequencing, which was repeated twice, did not clearly discriminate the SNP (Figure S2) and the corresponding genotype was separated as one haplotype. The difficulty in discriminating the SNP in Atlas 57 may reflect the use of seeds unintendedly mixed or the presence of heterozygosis. Only four barley genotypes showed the 1 kb insertion in the *HvAACT1* promoter and, in all of them, the 21 bp deletion and G-1,198 were detected (Table 1). The most common haplotype (corresponding to 41 genotypes) lacked the insertion in the *HvAACT1* promoter and had the 21 bp deletion and G-1,198. Interestingly, there was a high association between SNP-1,198 and the HvMATE-21indel marker. All accessions showing the 21 bp deletion also showed G-1,198 and, excluding Atlas 57, of the 30 genotypes with the 21 bp insertion, only four did not show T-1,198.

### Correlation between SNP and relative root length (RRL) in a short-term soil experiment

**Figure 4:**
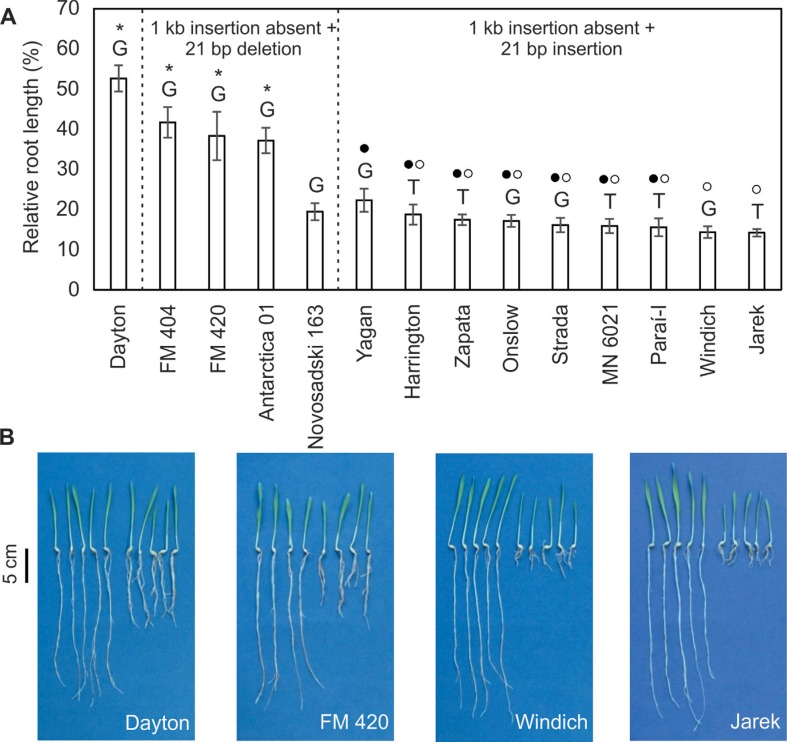
Relative root length (RRL) of 14 barley genotypes belonging to different haplotypes in a short-term soil experiment. **(A)** Relative root length expressed as a percentage. The columns represent the mean RRL ± SE_RRL_. Dayton was the only genotype to contain the 1 kb insertion upstream from the *HvAACT1* gene along with the 21 bp deletion of the HvMATE-21indel marker. Letters above each bar represent the SNP (T/G) at position 1,198 of the *HvAACT1* coding region. Asterisks indicate the RRLs that were significantly different (p < 0.05) from genotypes without the 1 kb insertion but containing the 21 bp insertion. Among the accessions lacking the 1 kb insertion and showing the 21 bp insertion, the filled dots indicate the RRL that were similar to Yagan and the open dots indicate the RRL that were similar to Jarek. **(B)** Plants (representatives of each haplotype) for which the roots were used to calculate the RRL. For each genotype, the plants on the left were grown on limed soil and those on the right were grown on acidic soil.

We ran a short-term soil experiment to calculate the relative root length (RRL) in 14 genotypes belonging to three haplotypes (Figure 4). There were differences in RRL among the genotypes such that the genotypes with a greater RRL always had G-1,198. However, the differences in RRL were not associated with SNP-1,198 and were better explained by the presence of the 1 kb insertion in the *HvAACT1* promoter and the alleles of the HvMATE-21indel marker. For instance, the genotype showing the 1 kb insertion in the *HvAACT1* promoter (Dayton), which also had the 21 bp deletion, had the highest RRL, while three (FM 404, FM 420 and Antarctica 01) of the four genotypes showing the 21 bp deletion also had a significantly greater RRL. Most of the accessions among the nine genotypes with the 21 bp insertion, but showing different SNPs (T or G-1,198), had a similar RRL (from 22.3 ± 2.9% in Yagan to 15.5 ± 2.2% in Paraí-I and from 18.7 ± 2.5% in Harrington to 14.2 ± 0.9% in Jarek). Only RRL for Yagan was significantly different from Windich and Jarek. Evaluation of these nine genotypes revealed the same allele for the 1 kb insertion, meaning that the major difference linked to the *HvAACT1* gene was related to the SNP. Thus, SNP G-1,198, which was previously associated with Al^3+^-tolerance (Figure S3), was not advantageous for better root growth on acidic soil when the same alleles for markers 1 kb insertion and HvMATE-21indel were present.

## Discussion

Understanding the molecular mechanisms that underlie Al^3+^-tolerance in plants is an important step for improving root growth on acidic soils/subsoils. The plant root is a key component in providing better yields (Den Herder *et al.*, 2010) and plants with deeper root growth are better suited to extract water and nutrients from deeper soil layers (Wasson *et al.*, 2012; Lynch and Wojciechowski, 2015). Moreover, roots from Al^3+^-tolerant plants are more efficient at taking up phosphorus from acid soil (Delhaize *et al.*, 2009). In barley, an increase in acidic soil tolerance is interesting since this is one of the most Al^3+^-sensitive plant species. In this work, we attempted to link SNPs detected in the *HvAACT1* coding region with barley root growth on acidic soil. We detected seven SNPs and one of them (T/G-1,198) was associated with Al^3+^-tolerance in contrasting genotypes (Figure S3). This SNP involved an L/V-172 substitution in the HvAACT1 protein and was the only difference between the HvAACT1 proteins of genotypes that differed in Al^3+^-tolerance (Figure S4). A high degree of similarity in the *HvAACT1* gene, with only four SNPs found in ten barley accessions and a difference of only two bases and one amino acid between Murasakimochi (Al^3+^-tolerant) and Morex (Al^3+^-sensitive), has previously been reported (Furukawa *et al.*, 2007). Moreover, alleles from two other organic acid transporter genes also showed high sequence similarity (Sasaki *et al.*, 2004; Tovkach *et al.*, 2013).

The HvAACT1 protein is an Al-activated citrate efflux transporter located in the plasma membrane and has seven putative transmembrane domains (Furukawa *et al.*, 2007). Our computational analysis done with two HvAACT1 proteins that differed only in residue 172 (L or V) revealed that both proteins were stable (Figure 2). However, the HvAACT1 protein with V-172, which was detected in genotypes with greater root growth on acidic soil, was more open at the lower end (Figures 3 and S5). Based on this observation, we hypothesized that this difference could facilitate the passage of citrate across the transporter, thus explaining the different performances of barley genotypes containing HvAACT1 proteins with V-172 or L-172. Clearly, a single amino acid substitution was enough to alter important phenotypic traits, as previously reported for barley (Chono *et al.*, 2003).

To determine whether the change detected in the computational analysis was biologically important, we measured root growth in genotypes with different SNPs (L or V-172). During the course of our investigation, this same SNP was reported for the *HvAACT1* gene in a comparison of the Brazilian Al^3+^-tolerant barley cultivar BR2 and the Australian Al^3+^-sensitive cultivar Hamelin (Bian *et al.*, 2015). These authors showed that the marker designed to detect the SNP co-segregated with Al^3+^-tolerance and accounted for 79% of the genetic variation for this trait. However, these two genotypes (BR2 and Hamelin) have different HvMATE-21indel alleles (Bian *et al.*, 2013). This raised the question as to whether the SNP itself was responsible for the differences in barley root growth on acidic soil or if the impact of the SNP was somehow associated with the 21 bp insertion/deletion located at the 3’ untranslated region of the *HvAACT1* gene. This uncertainty encouraged us to separate the genotypes into haplotypes based on the alleles of SNP-1,198 and the HvMATE-21indel marker and the presence of the 1 kb insertion in the HvAACT1 promoter. In this situation, the best way of evaluating the impact of the SNP was to compare genotypes with different SNPs (T or G-1,198) but with the same 1-kb insertion and HvMATE-21indel alleles. Haplotype-based phenotyping using a short-term soil experiment has also been used to evaluate the impact of different alleles of the wheat *TaMATE1B* gene, thereby allowing the comparison of genotypes with different *TaALMT1* alleles (Pereira *et al.*, 2015).

We evaluated 76 barley accessions that were separated in five haplotypes (Table 1). The insertion in the *HvAACT1* promoter was uncommon when compared to the absence of the insertion, a finding in agreement with Fujii *et al.* (2012) who detected this insertion in 20 out of 246 genotypes of cultivated barley. The most common haplotype that we detected lacked the insertion in the *HvAACT1* promoter and had the 21 bp deletion along with G-1,198 (53.9% of the genotypes). However, we were unable to identify genotypes with these same alleles for the 1 kb insertion and HvMATE-21indel markers but having T-1,198. In fact, all 45 genotypes showing the 21 bp deletion also had G-1,198, indicating a high association between them. Among the 30 genotypes lacking the 1 kb insertion but having the 21 bp insertion, only four (Onslow, Strada, Windich and Yagan) had T-1,198. When analyzed in the short-term soil experiment, most of the genotypes lacking the 1 kb insertion and having the 21 bp insertion, but showing different SNPs, performed similarly, indicating the low importance of this SNP for barley root growth on acidic soil. This means that the difference detected in our computational analysis, where the protein with V-172 appeared to have a more relaxed structure and hypothetically an increased citrate efflux, was not enough to generate differences in root growth. Most of the genotypes studied here showed V-172 together with a lack of the 1 kb insertion in the *HvAACT1* promoter (Table 1). The absence of this insertion was correlated with higher *HvAACT1* expression in the vascular bundle and lower expression in the cortex and root apices. This differential expression agreed with the primary function of this gene, namely, the loading of citrate into xylem for long-distance iron transport (Fujii *et al.*, 2012). Since no genotypes showed the 1 kb insertion and contrasting SNPs-1,198 we were unable to evaluate the impact of the SNP in barley genotypes showing higher expression of *HvAACT1* in the root apex.

Even though SNP-1,198 was not advantageous in our haplotype-based phenotyping, plant performance was associated with the presence of the insertion in the *HvAACT1* promoter and with the HvMATE-21indel marker (Figure 4). Significantly greater relative root growth was observed for Dayton, which has the insertion in the *HvAACT1* promoter and the 21 bp deletion for the marker HvMATE-21indel, and for FM 404, FM 420 and Antarctica 01 that also showed the 21 bp deletion but lacked the 1 kb insertion in the promoter. These markers appear to be the best ones for detecting Al^3+^-tolerant barley genotypes. For instance, all cultivars with the 1 kb insertion showed higher Al^3+^ tolerance compared to those without the insertion (Fujii *et al.*, 2012) and the HvMATE-21indel marker explained 66.9-71% of the variation in acid soil tolerance in barley (Bian *et al.*, 2013; Ma *et al.*, 2016).

In conclusion, we successfully detected SNP-1,198 in the *HvAACT1* coding region in barley genotypes that differed in Al^3+^ tolerance and our computational analysis suggested that there was structural variation between HvAACT1 proteins that differed only at residue 172 (L/V-172). However, most of the genotypes that were polymorphic for the SNP, but showed the same alleles for the 1 kb insertion in the *HvAACT1* promoter and the HvMATE-21indel marker, had similar root growth on acidic soil. SNP-1,198 was highly correlated with the HvMATE-21indel marker and all genotypes showing the 1 kb insertion also had G-1,198. Haplotype-based phenotyping was found to be more accurate for assessing the role of the SNP and the differences in barley root growth on acidic soil showed greater correlation with polymorphisms outside the coding region, such as those detected by the HvMATE-21indel marker and the presence/absence of the 1 kb insertion in the *HvAACT1* promoter.

## Supplementary material

The following online material is available for this article:

Figure S1 - Strategy for *HvAACT1* sequencing.

Figure S2 - Different alleles for the 1 kb insertion and HvMATE-21indel markers and SNPs detected at position 1,198 of the *HvAACT1* gene.

Figure S3 - Alignment of the *HvAACT1* gene from barley genotypes contrasting for Al^3+^ tolerance.

Figure S4 - Alignment of the HvAACT1 protein from barley genotypes contrasting for Al^3+^ tolerance.

Figure S5 - Changes in the structure of HvAACT1 proteins with L-172 or V-172 over time.
