# Supplementary material for: Is a non-synonymous SNP in the HvAACT1 coding region associated with acidic soil tolerance in barley?
Source: Genet Mol Biol. 2017 May 8;40(2):480–90. doi: 10.1590/1678-4685-GMB-2016-0225 (PMC5488463; doi:10.1590/1678-4685-GMB-2016-0225)
Supplement: Supplementary file 1 [file 1415-4757-gmb-1678-4685-GMB-2016-0225-Suppl01.pdf]

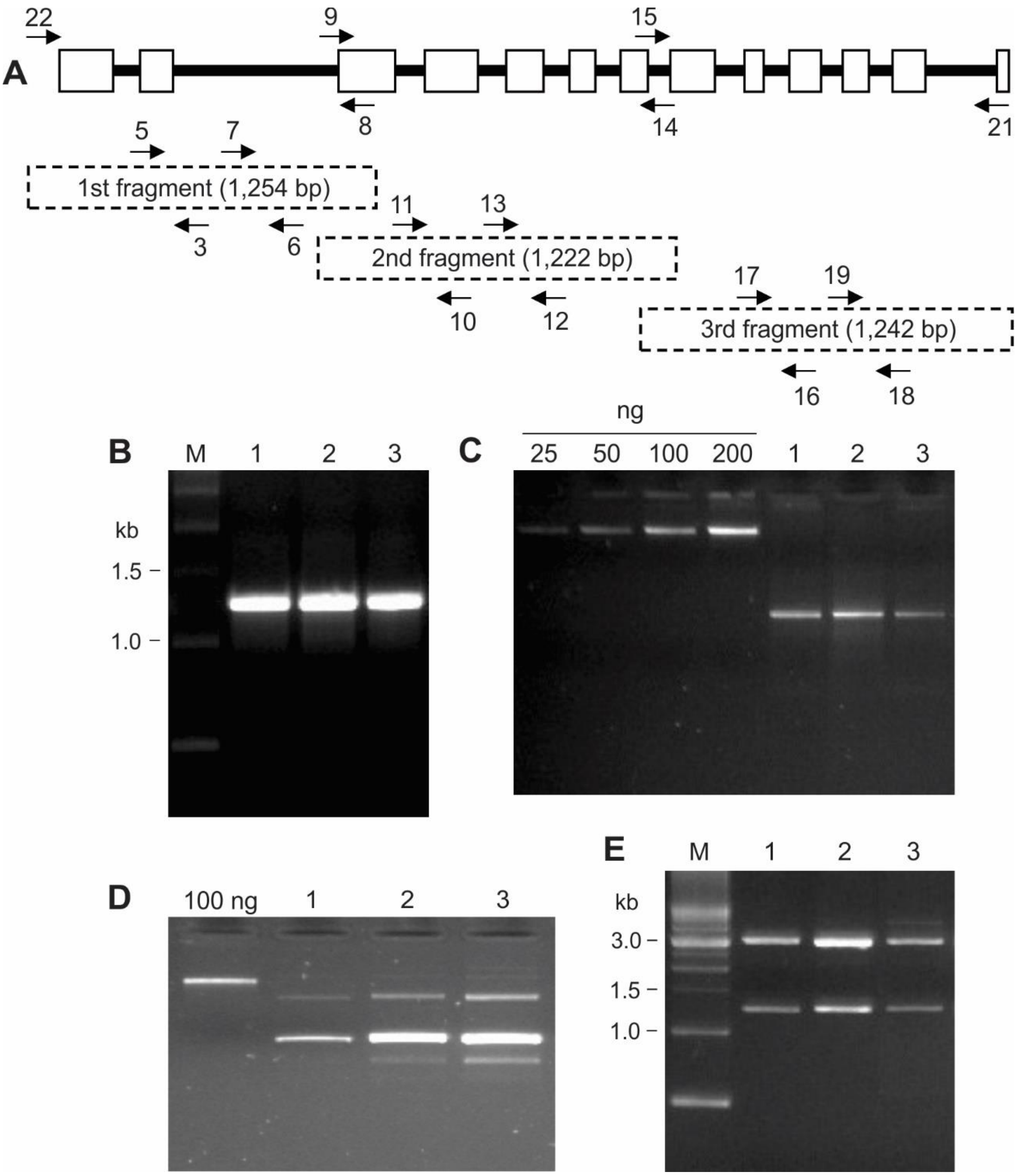

Figure S1 - Strategy for *HvAACT1* sequencing. **(A)** Structure of the *HvAACT1* gene where blocks indicate exons and line represent introns. Arrows indicate the primers used to obtain the 1st (primers 22 and 8), 2nd (primers 9 and 14) and 3rd (primers 15 and 21) fragments and for sequencing them. **(B)** Electrophoresis of the three fragments after PCR. **(C)** Quantification of the three fragments after precipitation. **(D)** Quantification of the purified plasmids. **(E)** Plasmids cut with *Eco*RI to confirm the cloning. The letter M indicates the molecular size marker while the numbers 1, 2 and 3 represent the 1st, 2nd and 3rd fragments, respectively.
