# Supplementary material for: Is a non-synonymous SNP in the HvAACT1 coding region associated with acidic soil tolerance in barley?
Source: Genet Mol Biol. 2017 May 8;40(2):480–90. doi: 10.1590/1678-4685-GMB-2016-0225 (PMC5488463; doi:10.1590/1678-4685-GMB-2016-0225)
Supplement: Supplementary file 2 [file 1415-4757-gmb-1678-4685-GMB-2016-0225-Suppl02.pdf]

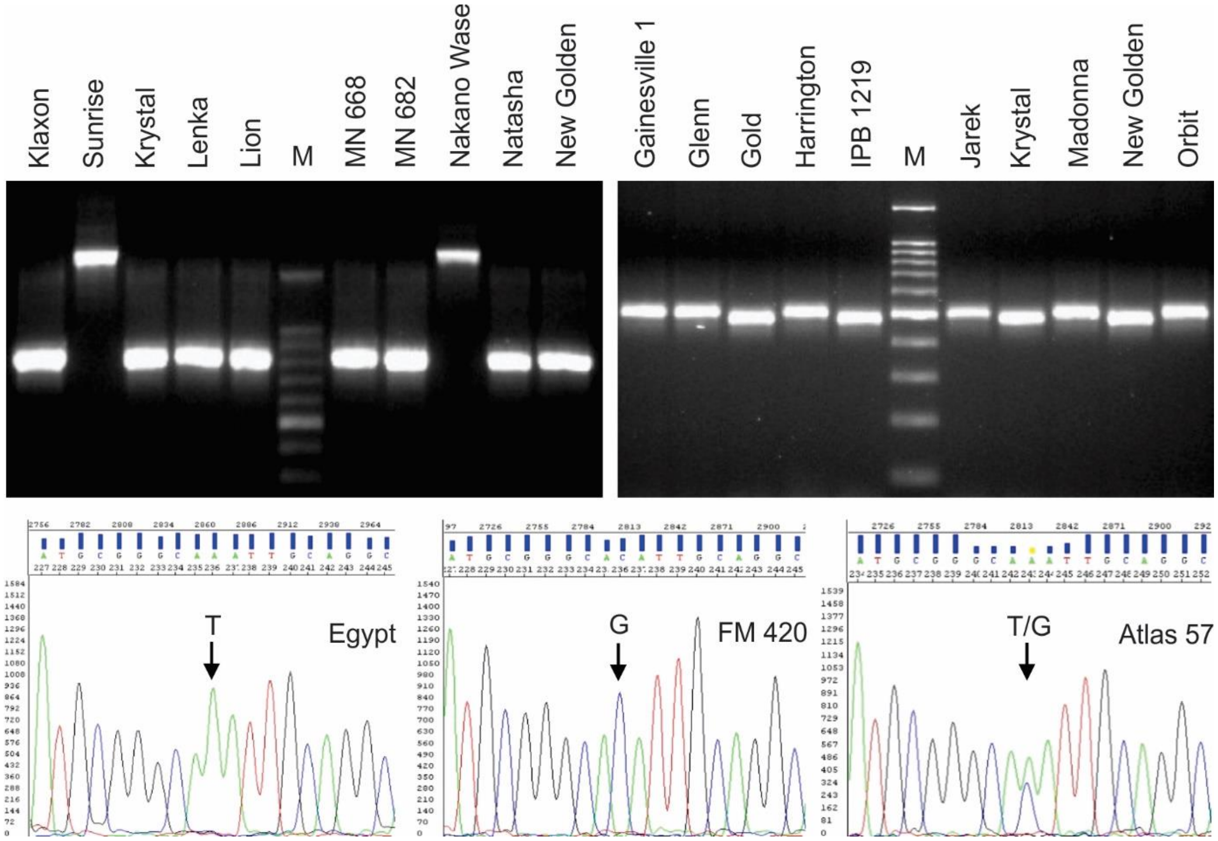

Figure S2 - Different alleles for the 1 kb insertion (*top left*) and HvMATE-21indel (*top right*) and SNPs detected at position 1,198 of the *HvAACT1* gene (*electropherograms at bottom*). Two amplicons were identified for the 1 kb insertion marker as described by Fujii et al. (2012): 1,844 bp indicating the presence of an insertion in the upstream region of the *HvAACT1* gene and 821 bp related to the absence of the insertion. Two fragments were amplified for marker HvMATE-21indel as described by Bian et al. (2013): 497 bp corresponding to a 21 bp deletion and 518 bp corresponding to a 21 bp insertion. '*M*' indicates the size marker used. The electropherograms indicate the sequences that allowed the identification of the SNPs (T/G-1,198) in the *HvAACT1* gene. The nucleotides above the arrows indicate bases as obtained in the reverse complementary sequence.
