# Supplementary material for: Is a non-synonymous SNP in the HvAACT1 coding region associated with acidic soil tolerance in barley?
Source: Genet Mol Biol. 2017 May 8;40(2):480–90. doi: 10.1590/1678-4685-GMB-2016-0225 (PMC5488463; doi:10.1590/1678-4685-GMB-2016-0225)
Supplement: Supplementary file 3 [file 1415-4757-gmb-1678-4685-GMB-2016-0225-Suppl03.pdf]

```
MN6021        ATGGAGGAGGGCGCGGCCGCGAGCATGATGACCGGGGACAAGAAGTGGGTGGCCGTCGTCGACGTCCCCGCAGACGCAGACGCTGCAACGGCGGCGAACGGACACGGCCCGGAGGAGAAGGCCGCGGAGGATCTGCCGGCGGCATTGTCCGGCTGCCCCAGGACGACGGGGCTCTACCTCTTTGTCAT
Paraí-I       ATGGAGGAGGGCGCGGCCGCGAGCATGATGACCGGGGACAAGAAGTGGGTGGCCGTCGTCGACGTCCCCGCAGACGCAGACGCTGCAACGGCGGCGAACGGACACGGCCCGGAGGAGAAGGCCGCGGAGGATCTGCCGGCGGCATTGTCCGGCTGCCCCAGGACGACGGGGCTCTACCTCTTTGTCAT
FM404         ATGGAGGAGGGCGCGGCCGCGAGCATGATGACCGGGGACAAGAAGTGGGTGGCCGTCGTCGACGTCCCCGCAGACGCAGACGCTGCAACGGCGGCGAACGGACACGGCCCGGAGGAGAAGGCCGCGGAGGATCTGCCGGCGGCATTGTCCGGCTGCCCCAGGACGACGGGGCTCTACCTCTTTGTCAT
Antarctica01  ATGGAGGAGGGCGCGGCCGCGAGCATGATGACCGGGGACAAGAAGTGGGTGGCCGTCGTCGACGTCCCCGCAGACGCAGACGCTGCAACGGCGGCGAACGGACACGGCCCGGAGGAGAAGGCCGCGGAGGATCTGCCGGCGGCATTGTCCGGCTGCCCCAGGACGACGGGGCTCTACCTCTTTGTCAT
              ********************************************************************************************************************************************************************************************

MN6021        GAACATCAGgttcgttgatttcctttcacatactgtacgtcttacgagtgctctcgtgttcgtcacgtctgatgtggtgaactgtaccttgtcgcagGAGCGTCTTCAAGCTCGACGAGCTCGGGTCGGAGGTGCTGCGCATTGCGGTGCCGGCGTCGCTTGCCCTGGCCGCCGATCCCCTGGCTTCC
Paraí-I       GAACATCAGgttcgttgatttcctttcacatactgtacgtcttacgagtgctctcgtgttcgtcacgtctgatgtggtgaactgtaccttgtcgcagGAGCGTCTTCAAGCTCGACGAGCTCGGGTCGGAGGTGCTGCGCATTGCGGTGCCGGCGTCGCTTGCCCTGGCCGCCGATCCCCTGGCTTCC
FM404         GAACATCAGgttcgttgatttcctttcacatactgtacgtcttacgagtgctctcgtgttcgtcacgtctgatgtggtgaactgtaccttgtcgcagGAGCGTCTTCAAGCTCGACGAGCTCGGGTCGGAGGTGCTGCGCATTGCGGTGCCGGCGTCGCTTGCCCTGGCCGCCGATCCCCTGGCTTCC
Antarctica01  GAACATCAGgttcgttgatttcctttcacatactgtacgtcttacgagtgctctcgtgttcgtcacgtctgatgtggtgaactgtaccttgtcgcagGAGCGTCTTCAAGCTCGACGAGCTCGGGTCGGAGGTGCTGCGCATTGCGGTGCCGGCGTCGCTTGCCCTGGCCGCCGATCCCCTGGCTTCC
              ********************************************************************************************************************************************************************************************

MN6021        TTGGTGGACACAGCATTCATCGGCCGTCTAGgtaatctcacgaccatccaaccaacatatctactatctgtttatgcaatgatgaagaagaaacatctatgccagtcgctgttgcagcaccatattcacctgcacctccccgtcttgcggaatttccgcgggatagataaagcttcccaattaatttt
Paraí-I       TTGGTGGACACAGCATTCATCGGCCGTCTAGgtaatctcacgaccatccaaccaacatatctactatctgtttatgcaatgatgaagaagaaacatctatgccagtcgctgttgcagcaccatattcacctgcacctccccgtcttgcggaatttccgcgggatagataaagcttcccaattaatttt
FM404         TTGGTGGACACAGCATTCATCGGCCGTCTAGgtaatctcacgaccatccaaccaacatatctactatctgtttatgcaatgatgaagaagaaacatctatgccagtcgctgttgcagcaccatattcacctgcacctccccgtcttgcggaatttccgcgggatagataaagcttcccaattaatttt
Antarctica01  TTGGTGGACACAGCATTCATCGGCCGTCTAGgtaatctcacgaccatccaaccaacatatctactatctgtttatgcaatgatgaagaagaaacatctatgccagtcgctgttgcagcaccatattcacctgcacctccccgtcttgcggaatttccgcgggatagataaagcttcccaattaatttt
              ********************************************************************************************************************************************************************************************

MN6021        caccggcggtcctaattttaggcagtcgtacaaggggatttccagatatgtgccaccttcgctgtttctgtcagtggatagtccccacttcagtttccggtttgtcagtttggcaaagtaactgacttcgctctacagtatcggtcgcttgatttggacatgtaaccggctcatttggcgccaaaaaa
Paraí-I       caccggcggtcctaattttaggcagtcgtacaaggggatttccagatatgtgccaccttcgctgtttctgtcagtggatagtccccacttcagtttccggtttgtcagtttggcaaagtaactgacttcgctctacagtatcggtcgcttgatttggacatgtaaccggctcatttggcgccaaaaaa
FM404         caccggcggtcctaattttaggcagtcgtacaaggggatttccagatatgtgccaccttcgctgtttctgtcagtggatagtccccacttcagtttccggtttgtcagtttggcaaagtaactgacttcgctctacagtatcggtcgcttgatttggacatgtaaccggctcatttggcgccaaaaaa
Antarctica01  caccggcggtcctaattttaggcagtcgtacaaggggatttccagatatgtgccaccttcgctgtttctgtcagtggatagtccccacttcagtttccggtttgtcagtttggcaaagtaactgacttcgctctacagtatcggtcgcttgatttggacatgtaaccggctcatttggcgccaaaaaa
              ********************************************************************************************************************************************************************************************

MN6021        ccttccacaaaatgcgcgcgggagaatttttcacctttgctgacaatagcgcccaccttatttttctgtttttgccaataggccatggctgataaattgctcatctagtgtatctccccgtccttctttctcctggtgaaacgggcatgtctctagtacatgtcttcatatgatatctttcattcttt
Paraí-I       ccttccacaaaatgcgcgcgggagaatttttcacctttgctgacaatagcgcccaccttatttttctgtttttgccaataggccatggctgataaattgctcatctagtgtatctccccgtccttctttctcctggtgaaacgggcatgtctctagtacatgtcttcatatgatatctttcattcttt
FM404         ccttccacaaaatgcgcgcgggagaatttttcacctttgctgacaatagcgcccaccttatttttctgtttttgccaataggccatggctgataaattgctcatctagtgtatctccccgtccttctttctcctggtgaaacgggcatgtctctagtacatgtcttcatatgatatctttcattcttt
Antarctica01  ccttccacaaaatgcgcgcgggagaatttttcacctttgctgacaatagcgcccaccttatttttctgtttttgccaataggccatggctgataaattgctcatctagtgtatctccccgtccttctttctcctggtgaaacgggcatgtctctagtacatgtcttcatatgatatctttcattcttt
              ********************************************************************************************************************************************************************************************

MN6021        cctttgatttggttattacattggtgttttcattaaatatttgcacaattggtccatgatcagGTTCGGTGGAGATAGCAGCTGTTGGTGTTTCTATTGCCATATTTAACCAAGTCTCCAAAGTCTGTATCTACCCGCTTGTTAGCGTAACAACATCATTCGTCGCTGAAGAAGATGCCATCATTAGC
Paraí-I       cctttgatttggttattacattggtgttttcattaaatatttgcacaattggtccatgatcagGTTCGGTGGAGATAGCAGCTGTTGGTGTTTCTATTGCCATATTTAACCAAGTCTCCAAAGTCTGTATCTACCCGCTTGTTAGCGTAACAACATCATTCGTCGCTGAAGAAGATGCCATCATTAGC
FM404         cctttgatttggttattacattggtgttttcattaaatatttgcacaattggtccatgatcagGTTCGGTGGAGATAGCAGCTGTTGGTGTTTCTATTGCCATATTTAACCAAGTCTCCAAAGTCTGTATCTACCCGCTTGTTAGCGTAACAACATCATTCGTCGCTGAAGAAGATGCCATCATTAGC
Antarctica01  cctttgatttggttattacattggtgttttcattaaatatttgcacaattggtccatgatcagGTTCGGTGGAGATAGCAGCTGTTGGTGTTTCTATTGCCATATTTAACCAAGTCTCCAAAGTCTGTATCTACCCGCTTGTTAGCGTAACAACATCATTCGTCGCTGAAGAAGATGCCATCATTAGC
              ********************************************************************************************************************************************************************************************

MN6021        AAATACCTAGAAGAAAATAGCAGCCAAGACCTTGAGAAAGCCTCTCATGTGCATTCAGATGCCTGCAATTTGCCCGCATCTGgtaccgagctcttgcaatatacctggtttcaaatttccaaatagtaatttgatttcaccccaggactgaagcattctgcatgaatctctatgtggaactctgtttt
Paraí-I       AAATACCTAGAAGAAAATAGCAGCCAAGACCTTGAGAAAGCCTCTCATGTGCATTCAGATGCCTGCAATTTGCCCGCATCTGgtaccgagctcttgcaatatacctggtttcaaatttccaaatagtaatttgatttcaccccaggactgaagcattctgcatgaatctctatgtggaactctgtttt
FM404         AAATACCTAGAAGAAAATAGCAGCCAAGACCTTGAGAAAGCCTCTCATGTGCATTCAGATGCCTGCAATGTGCCCGCATCTGgtaccgagctcttgcaatatacctggtttcaaatttccaaatagtaatttgatttcaccccaggactgaagcattctgcatgaatctctatgtggaactctgtttt
Antarctica01  AAATACCTAGAAGAAAATAGCAGCCAAGACCTTGAGAAAGCCTCTCATGTGCATTCAGATGCCTGCAATGTGCCCGCATCTGgtaccgagctcttgcaatatacctggtttcaaatttccaaatagtaatttgatttcaccccaggactgaagcattctgcatgaatctctatgtggaactctgtttt
              ********************************************************************* **********************************************************************************************************************

MN6021        acagGTCCTGATACGCCAGTGTGTGCTAATTCCTGCATACCCACAGAGTGTACTGATCTCTCAAATCAAGGGTGCAAGAAAAGGTACATACCTTCCGTGACATCTGCTCTAATCGTTGGCTCATTTCTCGGGCTAGTTCAGGCCGTGTTCCTGATCTTTTCGGCGAAATTCGTACTGGGCATCATGGG
Paraí-I       acagGTCCTGATACGCCAGTGTGCGCTAATTCCTGCATACCCACAGAGTGTACTGATCTCTCAAATCAAGGGTGCAAGAAAAGGTACATACCTTCCGTGACATCTGCTCTAATCGTTGGCTCATTTCTCGGGCTAGTTCAGGCCGTGTTCCTGATCTTTTCGGCGAAATTCGTACTGGGCATCATGGG
FM404         acagGTCCTGATACGCCAGTGTGTGCTAATTCCTGCATACCCACAGAGTGTACTGATCTCTCAAATCAAGGGTGCAAGAAAAGGTACATACCTTCCGTGACATCTGCTCTAATCGTTGGCTCATTTCTCGGGCTAGTTCAGGCCGTGTTCCTGATCTTTTCGGCGAAATTCGTACTGGGCATCATGGG
Antarctica01  acagGTCCTGATACGCCAGTGTGTGCTAATTCCTGCATACCCACAGAGTGTACTGATCTCTCAAATCAAGGGTGCAAGAAAAGGTACATACCTTCCGTGACATCTGCTCTAATCGTTGGCTCATTTCTCGGGCTAGTTCAGGCCGTGTTCCTGATCTTTTCGGCGAAATTCGTACTGGGCATCATGGG
              *********************** ********************************************************************************************************************************************************************

MN6021        TGTGAAACATgtaggtgttattaccttttgtatatgtccaagattattttacttctctagtttcttgctatccatgatgctaattcatattttcatctacatcacactattccagGACTCACCGATGCTAGAACCCGCGGTTCGATACCTAACGATCAGATCACTGGGCGCTCCTGCTGTTCTCCTGT
Paraí-I       TGTGAAACATgtaggtgttattaccttttgtatatgtccaagattattttacttctctagtttcttgctatccatgatgctaattcatattttcatctacatcacactattccagGACTCACCGATGCTAGAACCCGCGGTTCGATACCTAACGATCAGATCACTGGGCGCTCCTGCTGTTCTCCTGT
FM404         TGTGAAACATgtaggtgttattaccttttgtatatgtccaagattattttacttctctagtttcttgctatccatgatgctaattcatattttcatctacatcacactattccagGACTCACCGATGCTAGAACCCGCGGTTCGATACCTAACGATCAGATCACTGGGCGCTCCTGCTGTTCTCCTGT
Antarctica01  TGTGAAACATgtaggtgttattaccttttgtatatgtccaagattattttacttctctagtttcttgctatccatgatgctaattcatattttcatctacatcacactattccagGACTCACCGATGCTAGAACCCGCGGTTCGATACCTAACGATCAGATCACTGGGCGCTCCTGCTGTTCTCCTGT
              ********************************************************************************************************************************************************************************************

MN6021        CTTTGGCAATGCAGGGCGTTTTCCGGGGCTTCAAAGATACAAAGACACCGTTGTATGCTACTGgtaagaatatctggtgatgtcctatataggtttctgtttttaggaagaggataattgaagagaagcatcactgacatttccttacaatttcttttgcagTGGTTGGGGATGCAACAAATATCATC
Paraí-I       CTTTGGCAATGCAGGGCGTTTTCCGGGGCTTCAAAGATACAAAGACACCGTTGTATGCTACTGgtaagaatatctggtgatgtcctatataggtttctgtttttaggaagaggataattgaagagaagcatcactgacatttccttacaatttcttttgcagTGGTTGGGGATGCAACAAATATCATC
FM404         CTTTGGCAATGCAGGGCGTTTTCCGGGGCTTCAAAGATACAAAGACACCGTTGTATGCTACTGgtaagaatatctggtgatgtcctatataggtttctgtttttaggaagaggataattgaagagaagcatcactgacatttccttacaatttcttttgcagTGGTTGGGGATGCAACAAATATCATC
Antarctica01  CTTTGGCAATGCAGGGCGTTTTCCGGGGCTTCAAAGATACAAAGACACCGTTGTATGCTACTGgtaagaatatctggtgatgtcctatataggtttctgtttttaggaagaggataattgaagagaagcatcactgacatttccttacaatttcttttgcagTGGTTGGGGATGCAACAAATATCATC
              ********************************************************************************************************************************************************************************************

MN6021        CTAGATCCAATTTTGATGTTTGTCTGCCACATGGGTGTCACTGGTGCAGCAGTTGCTCATGTCATTTCCCAgtaggttttaccgctacaattagcatctgcagcctatgtgtttcagtgatcagtctgtcctaacaacgctccattccatcttttgccttcagGTACCTGATAACTATGATCTTGATA
Paraí-I       CTAGATCCAATTTTGATGTTTGTCTGCCACATGGGTGTCACTGGTGCAGCAGTTGCTCATGTCATTTCCCAgtaggttttaccgctacaattagcatctgcagcctatgtgtttcagtgatcagtctgtcctaacaacgctccattccatcttttgccttcagGTACCTGATAACTATGATCTTGATA
FM404         CTAGATCCAATTTTGATGTTTGTCTGCCACATGGGTGTCACTGGTGCAGCAGTTGCTCATGTCATTTCCCAgtaggttttaccgctacaattagcatctgcagcctatgtgtttcagtgatcagtctgtcctaacaacgctccattccatcttttgccttcagGTACCTGATAACTATGATCTTGATA
Antarctica01  CTAGATCCAATTTTGATGTTTGTCTGCCACATGGGTGTCACTGGTGCAGCAGTTGCTCATGTCATTTCCCAgtaggttttaccgctacaattagcatctgcagcctatgtgtttcagtgatcagtctgtcctaacaacgctccattccatcttttgccttcagGTACCTGATAACTATGATCTTGATA
              ********************************************************************************************************************************************************************************************

MN6021        TGTCGGCTCGTCCAGCAAGTTGATGTTATCCCACCGAGCCTTAAATCCCTGAAATTTGGGCGGTTTCTTGGTTGTGgtgagatactgttggaatcctgaaactggataacatctctcactggtcccctggaaatggctctgaaaatgctctgttttcttcagGATTCCTGCTGCTCGCAAGGGTGGTA
Paraí-I       TGTCGGCTCGTCCAGCAAGTTGATGTTATCCCACCGAGCCTTAAATCCCTGAAATTTGGGCGGTTTCTTGGTTGTGgtgagatactgttggaatcctgaaactggataacatctctcactggtcccctggaaatggctctgaaaatgctctgttttcttcagGATTCCTGCTGCTCGCAAGGGTGGTA
FM404         TGTCGGCTCGTCCAGCAAGTTGATGTTATCCCACCGAGCCTTAAATCCCTGAAATTTGGGCGGTTTCTTGGTTGTGgtgagatactgttggaatcctgaaactggataacatctctcactggtcccctggaaatggctctgaaaatgctctgttttcttcagGATTCCTGCTGCTCGCAAGGGTGGTA
Antarctica01  TGTCGGCTCGTCCAGCAAGTTGATGTTATCCCACCGAGCCTTAAATCCCTGAAATTTGGGCGGTTTCTTGGTTGTGgtgagatactgttggaatcctgaaactggataacatctctcactggtcccctggaaatggctctgaaaatgctctgttttcttcagGATTCCTGCTGCTCGCAAGGGTGGTA
              ********************************************************************************************************************************************************************************************
```

MN6021	GCGGTGACGTTCTGTGTCACTCTGGCGTCGTCGCTGGCTGCCCGCGACGGACCTACCATCATGGCAGCCTTCCAGATCTGCTGCCAGCTCTGGCTCGCGACGTCACTTCTCGCCGATGGATTGGCCGTTGCTGGACAG*gtcagaccgccaaatcatattcccatatatttattggtccatgaggaaca*
Paraí-I	GCGGTGACGTTCTGTGTCACTCTGGCGTCGTCGCTGGCTGCCCGCGACGGACCTACCATCATGGCGGCCTTCCAGATCTGCTGCCAGCTCTGGCTCGCGACGTCACTTCTCGCCGATGGATTGGCCGTTGCTGGACAG*gtcagaccgccaaatcatattcccatatatttattggtccatgaggaaca*
FM404	GCGGTGACGTTCTGTGTCACTCTGGCGTCGTCGCTGGCTGCCCGCGACGGACCTACCATCATGGCGGCCTTCCAGATCTGCTGCCAGCTCTGGCTCGCGACGTCACTTCTCGCCGATGGATTGGCCGTTGCTGGACAG*gtcagaccgccaaatcatattcccatatatttattggtccatgaggaaca*
Antarctica01	GCGGTGACGTTCTGTGTCACTCTGGCATCGTCGCTGGCTGCCCGCGACGGACCTACCATCATGGCGGCCTTCCAGATCTGCTGCCAGCTCTGGCTCGCGACGTCACTTCTCGCCGATGGATTGGCCGTTGCTGGACAG*gtcagaccgccaaatcatattcccatatatttattggtccatgaggaaca*
	************************** ************************************** **
MN6021	*gcttcagttgagactagagagtgtgctgaactggggtgtgttgcggatgagttcttgcag*GCAGTGCTCGCAAGCGCGTTCGCCAAGAACGATCACAAGAAGGTGATTGCCGCGACCTCTCGTGTTCTGCAG*gtttgtgaccgttgattcttgccacttctccatatatttttcccagagtttcaatt*
Paraí-I	*gcttcagttgagactagagagtgtgctgaactggggtgtgttgcggatgagttcttgcag*GCAGTGCTCGCAAGCGCGTTCGCCAAGAACGATCACAAGAAGGTGATTGCCGCGACCTCTCGTGTTCTGCAG*gtttgtgaccgttgattcttgccacttctccatatatttttcccagagtttcaatt*
FM404	*gcttcagttgagactagagagtgtgctgaactggggtgtgttgcggatgagttcttgcag*GCAGTGCTCGCAAGCGCGTTCGCCAAGAACGATCACAAGAAGGTGATTGCCGCGACCTCTCGTGTTCTGCAG*gtttgtgaccgttgattcttgccacttctccatatatttttcccagagtttcaatt*
Antarctica01	*gcttcagttgagactagagagtgtgctgaactggggtgtgttgcggatgagttcttgcag*GCAGTGCTCGCAAGCGCGTTCGCCAAGAACGATCACAAGAAGGTGATTGCCGCGACCTCTCGTGTTCTGCAG*gtttgtgaccgttgattcttgccacttctccatatatttttcccagagtttcaatt*
	**
MN6021	*cattgagaagagttaatggtgacctctttctctttgcag*CTGAGCATCGTTCTGGGGATGGGCCTGACGGTGGTGCTTGGTCTCTTCATGAAGTTCGGCGCTGGCGTTTTCACGAGGGACGCAGACGTGATCAACGTCATCCACAAAGGCATCCCG*gtaagaaaccctggctgtacaaaacatgagaa*
Paraí-I	*cattgagaagagttaatggtgacctctttctctttgcag*CTGAGCATCGTTCTGGGGATGGGCCTGACGGTGGTGCTTGGTCTCTTCATGAAGTTCGGCGCTGGCGTTTTCACGAGGGACGCAGACGTGATCAACGTCATCCACAAAGGCATCCCG*gtaagaaaccttggctgtacaaaacatgagaa*
FM404	*cattgagaagagttaatggtgacctctttctctttgcag*CTGAGCATCGTTCTGGGGATGGGCCTGACGGTGGTGCTTGGTCTCTTCATGAAGTTCGGCGCTGGCGTTTTCACGAGGGACGCAGACGTGATCAACGTCATCCACAAAGGCATCCCG*gtaagaaaccttggctgtacaaaacatgagaa*
Antarctica01	*cattgagaagagttaatggtgacctctttctctttgcag*CTGAGCATCGTTCTGGGGATGGGCCTGACGGTGGTGCTTGGTCTCTTCATGAAGTTCGGCGCTGGCGTTTTCACGAGGGACGCAGACGTGATCAACGTCATCCACAAAGGCATCCCG*gtaagaaaccttggccgtacaaaacatgagaa*
	** **** ****************
MN6021	*tgccatacgagattcagacactgaaaatagctctgtttgggggttgttcag*TTTGTCGCCGGCACGCAGACGATAAACGCCCTCGCGTTCGTGTTCGACGGCATCAACTTCGGAGCACAAGACTACACCTACTCTGCATACTCCATG*gtaccgtagaccctttcacagcagcatcaatattttagctc*
Paraí-I	*tgccatacgagattcagacactgaaaatagctctgtttgggggttgttcag*TTTGTCGCCGGCACGCAGACGATAAACGCCCTCGCGTTCGTGTTCGACGGCATCAACTTCGGAGCACAAGACTACACCTACTCTGCATACTCCATG*gtaccgtagaccctttcacagcagcatcaatattttagctc*
FM404	*tgccatacgagattcagacactgaaaatagctctgtttgggggttgttcag*TTTGTCGCCGGCACGCAGACGATAAACGCCCTCGCGTTCGTGTTCGACGGCATCAACTTCGGAGCACAAGACTACACCTACTCTGCATACTCCATG*gtaccgtagaccctttcacagcagcatcaatattttagctc*
Antarctica01	*tgccatacgagattcagacactgaaaatagctctgtttgggggttgttcag*TTTGTCGCCGGCACGCAGACGATAAACGCCCTCGCGTTCGTGTTCGATGGCATCAACTTCGGAGCACAAGACTACACCTACTCTGCATACTCCATG*gtaccgtagaccctttcacagcagcatcaatattttagctc*
	** ***
MN6021	*tgaaacttgtcccgagactgaaattggatcccatggaaaccttcag*GTTGGGGTGGCGTCCATATCGATACCGTGCCTGGTGTACCTCTCGGCGCACAAGGGATTCATCGGCATATGGGTCGCATTGACCATCTACATGAGCCTCAGGACCGTAGCTAGCACCTGGAG*gtatgaacgatactatgctc*
Paraí-I	*tgaaacttgtcccgagactgaaattggatcccatggaaaccttcag*GTTGGGGTGGCGTCCATATCGATACCGTGCCTGGTGTACCTCTCGGCGCACAAGGGATTCATCGGCATATGGGTCGCATTGACCATCTACATGAGCCTCAGGACCGTAGCTAGCACCTGGAG*gtatgaacgatactatgctc*
FM404	*tgaaacttgtcccgagactgaaattggatcccatggaaaccttcag*GTTGGGGTGGCGTCCATATCGATACCGTGCCTGGTGTACCTCTCGGCGCACAAGGGATTCATCGGCATATGGGTCGCATTGACCATCTACATGAGCCTCAGGACCGTAGCTAGCACCTGGAG*gtatgaacgatactatgctc*
Antarctica01	*tgaaacttgtcccgagactgaaattggatcccatggaaaccttcag*GTTGGGGTGGCGTCCATATCGATACCGTGCCTGGTGTACCTCTCGGCGCACAAGGGATTCATCGGCATATGGGTCGCATTGACCATCTACATGAGCCTCAGGACCGTAGCTAGCACCTGGAG*gtatgaacgatactatgctc*
	**
MN6021	*ctccaccctacccccaccccccctcctcatcacttcatcagtacctccatttcttcggtagccccgaccccttttgtagcgccgctgacaggaggaatgttttcaaatcctgctgaaattgaactgttctatgtgaaacccttgcaaaatatgtgttctataatcttcaattggggctcatcaaggttt*
Paraí-I	*ctccaccctacccccaccccccctcctcatcacttcatcagtacctccatttcttcggtagccccgaccccttttgtagcgccgctgacaggaggaatgttttcaaatcctgctgaaattgaactgttctatgtgaaacccttgcaaaatatgtgttctataatcttcaattggggctcatcaaggttt*
FM404	*ctccaccctacccccaccccccctcctcatcacttcatcagtacctccatttcttcggtagccccgaccccttttgtagcgccgctgacaggaggaatgttttcaaatcctgctgaaattgaactgttctatgtgaaacccttgcaaaatatgtgttctataatcttcaattggggctcatcaaggttt*
Antarctica01	*ctccaccctacccccaccccccctcctcatcacttcatcagtacctccatttcttcggtagccccgaccccttttgtagcgccgctgacaggaggaatgttttcaaatcctgctgaaattgaactgttctatgtgaaacccttgcaaaatatgtgttctataatcttcaattggggctcatcaaggttt*
	**
MN6021	*tgtgactctggtgtctgcag*GATGGGGGCTGCGAGAGGGCCATGGGTTTTCCTCCGGAAG**TGA**
Paraí-I	*tgtgactctggtgtctgcag*GATGGGGGCTGCGAGAGGGCCATGGGTTTTCCTCCGGAAG**TGA**
FM404	*tgtgactctggtgtctgcag*GATGGGGGCTGCGAGAGGGCCATGGGTTTTCCTCCGGAAG**TGA**
Antarctica01	*tgtgactctggtgtctgcag*GATGGGGGCTGCGAGAGGGCCATGGGTTTTCCTCCGGAAG**TGA**
	***

Figure S3 - Alignment of *HvAACT1* gene from barley genotypes contrasting for Al^{3+} tolerance (MN 6021 and Paraí-I show lower root growth on acidic soil while FM404 and Antarctica 01 present higher root growth). Asterisks represent common bases among the sequences while bases highlighted in gray are different. Initiation and termination codons are bold. Uppercase letters represent exons and lowercase and italic letters represent intros. GenBank accessions numbers are KX278715 (MN 6021), KX278716 (Paraí-I), KX278714 (FM404) and KX278713 (Antarctica 01).
