# Supplementary material for: Is a non-synonymous SNP in the HvAACT1 coding region associated with acidic soil tolerance in barley?
Source: Genet Mol Biol. 2017 May 8;40(2):480–90. doi: 10.1590/1678-4685-GMB-2016-0225 (PMC5488463; doi:10.1590/1678-4685-GMB-2016-0225)
Supplement: Supplementary file 4 [file 1415-4757-gmb-1678-4685-GMB-2016-0225-Suppl04.pdf]

```
MN6021          MEEGAAASMMTGDKKWVAVVDVPADADAATAANGHGPEEKAAEDLPAALSGCPRTTGLYLFVMNIRSVFKLDELG
Parai           MEEGAAASMMTGDKKWVAVVDVPADADAATAANGHGPEEKAAEDLPAALSGCPRTTGLYLFVMNIRSVFKLDELG
Haruna Nijo     MEEGAAASMMTGDKKWVAVVDVPADADAATAANGHGPEEKAAEDLPAALSGCPRTTGLYLFVMNIRSVFKLDELG
Murasakimochi   MEEGAAASMMTGDKKWVAVVDVPADADAATAANGHGPEEKAAEDLPAALSGCPRTTGLYLFVMNIRSVFKLDELG
Antarctica01    MEEGAAASMMTGDKKWVAVVDVPADADAATAANGHGPEEKAAEDLPAALSGCPRTTGLYLFVMNIRSVFKLDELG
FM404           MEEGAAASMMTGDKKWVAVVDVPADADAATAANGHGPEEKAAEDLPAALSGCPRTTGLYLFVMNIRSVFKLDELG
                ***************************************************************************

MN6021          SEVLRIAVPASLALAADPLASLVDTAFIGRLGSVEIAAVGVSIAIFNQVSKVCIYPLVSVTTSFVAEEDAIISKY
Parai           SEVLRIAVPASLALAADPLASLVDTAFIGRLGSVEIAAVGVSIAIFNQVSKVCIYPLVSVTTSFVAEEDAIISKY
Haruna Nijo     SEVLRIAVPASLALAADPLASLVDTAFIGRLGSVEIAAVGVSIAIFNQVSKVCIYPLVSVTTSFVAEEDAIISKY
Murasakimochi   SEVLRIAVPASLALAADPLASLVDTAFIGRLGSVEIAAVGVSIAIFNQVSKVCIYPLVSVTTSFVAEEDAIISKY
Antarctica01    SEVLRIAVPASLALAADPLASLVDTAFIGRLGSVEIAAVGVSIAIFNQVSKVCIYPLVSVTTSFVAEEDAIISKY
FM404           SEVLRIAVPASLALAADPLASLVDTAFIGRLGSVEIAAVGVSIAIFNQVSKVCIYPLVSVTTSFVAEEDAIISKY
                ***************************************************************************

MN6021          LEENSSQDLEKASHVHSDACNLPASGPDTPVCANSCIPTECTDLSNQGCKKRYIPSVTSALIVGSFLGLVQAVFL
Parai           LEENSSQDLEKASHVHSDACNLPASGPDTPVCANSCIPTECTDLSNQGCKKRYIPSVTSALIVGSFLGLVQAVFL
Haruna Nijo     LEENSSQDLEKASHVHSDACNLPASGPDTPVCANSCIPTECTDLSNQGCKKRYIPSVTSALIVGSFLGLVQAVFL
Murasakimochi   LEENSSQDLEKASHVHSDACNVPASGPDTPVCANSCIPTECTDLSNQGCKKRYIPSVTSALIVGSFLGLVQAVFL
Antarctica01    LEENSSQDLEKASHVHSDACNVPASGPDTPVCANSCIPTECTDLSNQGCKKRYIPSVTSALIVGSFLGLVQAVFL
FM404           LEENSSQDLEKASHVHSDACNVPASGPDTPVCANSCIPTECTDLSNQGCKKRYIPSVTSALIVGSFLGLVQAVFL
                *********************:*****************************************************

MN6021          IFSAKFVLGIMGVKHDSPMLEPAVRYLTIRSLGAPAVLLSLAMQGVFRGFKDTKTPLYATVVGDATNIILDPILM
Parai           IFSAKFVLGIMGVKHDSPMLEPAVRYLTIRSLGAPAVLLSLAMQGVFRGFKDTKTPLYATVVGDATNIILDPILM
Haruna Nijo     IFSAKFVLGIMGVKHDSPMLEPAVRYLTIRSLGAPAVLLSLAMQGVFRGFKDTKTPLYATVVGDATNIILDPILM
Murasakimochi   IFSAKFVLGIMGVKHDSPMLEPAVRYLTIRSLGAPAVLLSLAMQGVFRGFKDTKTPLYATVVGDATNIILDPILM
Antarctica01    IFSAKFVLGIMGVKHDSPMLEPAVRYLTIRSLGAPAVLLSLAMQGVFRGFKDTKTPLYATVVGDATNIILDPILM
FM404           IFSAKFVLGIMGVKHDSPMLEPAVRYLTIRSLGAPAVLLSLAMQGVFRGFKDTKTPLYATVVGDATNIILDPILM
                ***************************************************************************

MN6021          FVCHMGVTGAAVAHVISQYLITMILICRLVQQVDVIPPSLKSLKFGRFLGCGFLLLARVVAVTFCVTLASSLAAR
Parai           FVCHMGVTGAAVAHVISQYLITMILICRLVQQVDVIPPSLKSLKFGRFLGCGFLLLARVVAVTFCVTLASSLAAR
Haruna Nijo     FVCHMGVTGAAVAHVISQYLITMILICRLVQQVDVIPPSLKSLKFGRFLGCGFLLLARVVAVTFCVTLASSLAAR
Murasakimochi   FVCHMGVTGAAVAHVISQYLITMILICRLVQQVDVIPPSLKSLKFGRFLGCGFLLLARVVAVTFCVTLASSLAAR
Antarctica01    FVCHMGVTGAAVAHVISQYLITMILICRLVQQVDVIPPSLKSLKFGRFLGCGFLLLARVVAVTFCVTLASSLAAR
FM404           FVCHMGVTGAAVAHVISQYLITMILICRLVQQVDVIPPSLKSLKFGRFLGCGFLLLARVVAVTFCVTLASSLAAR
                ***************************************************************************

MN6021          DGPTIMAAFQICCQLWLATSLLADGLAVAGQAVLASAFAKNDHKKVIAATSRVLQLSIVLGMGLTVVLGLFMKFG
Parai           DGPTIMAAFQICCQLWLATSLLADGLAVAGQAVLASAFAKNDHKKVIAATSRVLQLSIVLGMGLTVVLGLFMKFG
Haruna Nijo     DGPTIMAAFQICCQLWLATSLLADGLAVAGQAVLASAFAKNDHKKVIAATSRVLQLSIVLGMGLTVVLGLFMKFG
Murasakimochi   DGPTIMAAFQICCQLWLATSLLADGLAVAGQAVLASAFAKNDHKKVIAATSRVLQLSIVLGMGLTVVLGLFMKFG
Antarctica01    DGPTIMAAFQICCQLWLATSLLADGLAVAGQAVLASAFAKNDHKKVIAATSRVLQLSIVLGMGLTVVLGLFMKFG
FM404           DGPTIMAAFQICCQLWLATSLLADGLAVAGQAVLASAFAKNDHKKVIAATSRVLQLSIVLGMGLTVVLGLFMKFG
                ***************************************************************************

MN6021          AGVFTRDADVINVIHKGIPFVAGTQTINALAFVFDGINFGAQDYTYSAYSMVGVASISIPCLVYLSAHKGFIGIW
Parai           AGVFTRDADVINVIHKGIPFVAGTQTINALAFVFDGINFGAQDYTYSAYSMVGVASISIPCLVYLSAHKGFIGIW
Haruna Nijo     AGVFTRDADVINVIHKGIPFVAGTQTINALAFVFDGINFGAQDYTYSAYSMVGVASISIPCLVYLSAHKGFIGIW
Murasakimochi   AGVFTRDADVINVIHKGIPFVAGTQTINALAFVFDGINFGAQDYTYSAYSMVGVASISIPCLVYLSAHKGFIGIW
Antarctica01    AGVFTRDADVINVIHKGIPFVAGTQTINALAFVFDGINFGAQDYTYSAYSMVGVASISIPCLVYLSAHKGFIGIW
FM404           AGVFTRDADVINVIHKGIPFVAGTQTINALAFVFDGINFGAQDYTYSAYSMVGVASISIPCLVYLSAHKGFIGIW
                ***************************************************************************

MN6021          VALTIYMSLRTVASTWRMGAARGPWVFLRK
Parai           VALTIYMSLRTVASTWRMGAARGPWVFLRK
Haruna Nijo     VALTIYMSLRTVASTWRMGAARGPWVFLRK
Murasakimochi   VALTIYMSLRTVASTWRMGAARGPWVFLRK
Antarctica01    VALTIYMSLRTVASTWRMGAARGPWVFLRK
FM404           VALTIYMSLRTVASTWRMGAARGPWVFLRK
                ******************************
```

Figure S4 - Alignment of the HvAACT1 protein from barley genotypes contrasting for Al^{3+} tolerance. Aminoacids highlighted in gray are different. GenBank accession numbers for Al^{3+} sensitive genotypes are KX278715 (MN 6021), KX278716 (Paraí-I) and BAF75823 (Haruna Nijo). GenBank accession numbers for Al^{3+} tolerant genotypes are BAF75822 (Murasakimochi), KX278713 (Antarctica 01) and KX278714 (FM 404).
