# Supplementary material for: Is a non-synonymous SNP in the HvAACT1 coding region associated with acidic soil tolerance in barley?
Source: Genet Mol Biol. 2017 May 8;40(2):480–90. doi: 10.1590/1678-4685-GMB-2016-0225 (PMC5488463; doi:10.1590/1678-4685-GMB-2016-0225)
Supplement: Supplementary file 5 [file 1415-4757-gmb-1678-4685-GMB-2016-0225-Suppl05.pdf]

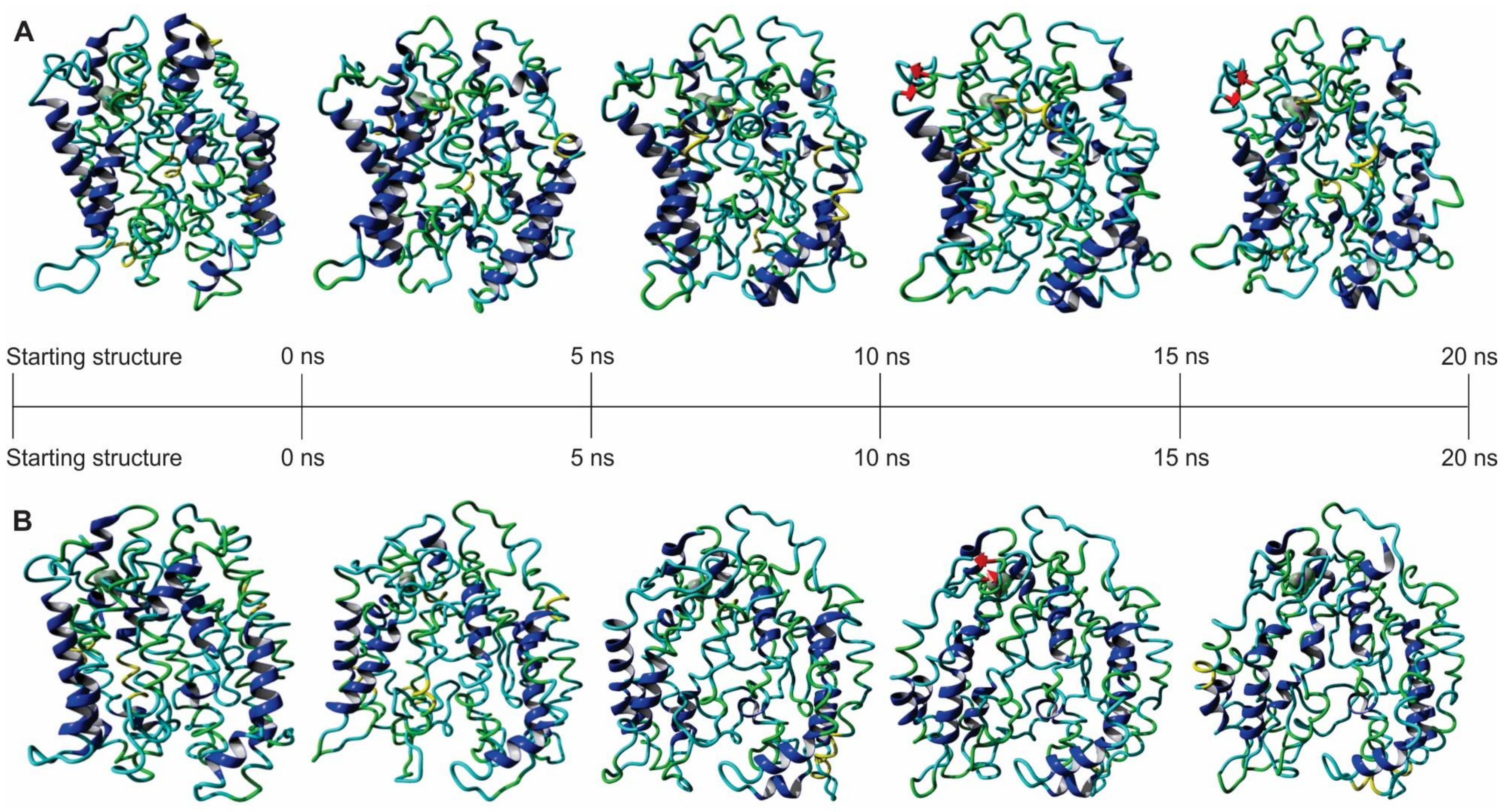

Figure S5 - Changes in the structure of HvAACT1 proteins with L-172 **(A)** or V-172 **(B)** over time (0 to 20 ns). Grey cloud represents the position of residue 172.
